# Symptom management, nutrition and hydration at end-of-life: a qualitative exploration of patients’, carers’ and health professionals’ experiences and further research questions

**DOI:** 10.1186/s12904-018-0314-4

**Published:** 2018-04-16

**Authors:** Jessica Baillie, Despina Anagnostou, Stephanie Sivell, Jordan Van Godwin, Anthony Byrne, Annmarie Nelson

**Affiliations:** 1 School of Healthcare Sciences, Cardiff University, Cardiff, UK; 2 Marie Curie Palliative Care Research Centre, Division of Population Medicine, School of Medicine, Cardiff University, Cardiff, UK; 3 DECIPHer, School of Social Sciences, Cardiff University, Cardiff, UK

**Keywords:** Symptom assessment, Pain management, Nutritional status, Dehydration, Palliative care, Terminal care, Qualitative research

## Abstract

**Background:**

Symptom management is an essential aspect of palliative and end-of-life care, but evidence suggests that patients’ symptoms may not always be relieved, causing significant harm to patients and magnifying their relatives’ distress. A growing body of evidence focuses on symptom management at the end-of-life, but research funding for palliative care remains disproportionately low. It is therefore crucial that research funding is targeted at areas of importance to patients and relatives. The Palliative and end-of-life care Priority Setting Partnership (PeolcPSP) undertook a UK-wide free-text survey to establish research priorities within palliative and end-of-life care and disseminated its results in 2015. Much of the data were related more broadly to personal perceptions and experiences rather than specific research questions. The aim of this article is to report on a supplementary analysis exploring the experiences and questions of PeolcPSP survey respondents regarding symptoms, hydration and nutrition.

**Methods:**

The PeolcPSP data (*n* = 1403) were coded by a team of qualitative researchers in a supplementary analysis. There were 190 responses that related to symptoms, nutrition and hydration. The data were analysed thematically using Braun and Clarke’s approach.

**Results:**

Five themes were identified: pain, breathlessness, agitation, nutrition and hydration. The majority of responses related to symptoms that were sub-optimally managed, in particular pain. Nutrition and hydration were of significant concern, particularly for carers. Overall, respondents consistently asked about the most effective, evidence-based methods for managing symptoms and suggested areas where further research is necessary.

**Conclusions:**

This study highlights the perceptions and experiences of patients, families and professionals within palliative care, highlighting the need for improved care, communication and further research to establish which treatments are most effective within a palliative care population. This is essential to reduce harm and distress for patients and families.

**Electronic supplementary material:**

The online version of this article (10.1186/s12904-018-0314-4) contains supplementary material, which is available to authorized users.

## Background

The World Health Organisation estimates that 20 million people need palliative care around the world each year [1]. In high income countries, such as the United Kingdom (UK), 69–82% of people who die need palliative care [2]. Furthermore, a recent analysis suggests that by 2040, 87.6% of dying people will need palliative care [3]. The palliative care approach aims to improve the “quality of life of patients and their families facing the problem associated with life-threatening illness” [4]. Access to specialist palliative care has been found to increase likelihood of dying in the preferred place of care, is economically more effective and reduces symptom burden [5].

Management of symptoms, including pain, is an essential aspect of palliative care, along with psychological, spiritual and social support [4]. A recent systematic review of 143 studies of people with malignant and non-malignant conditions, identified that the following symptoms had 50% or more prevalence: pain, fatigue, anorexia, dyspnoea and worry [6]. Management of symptoms is considered a priority by relatives of people at the end of their lives [7], however patients’ symptoms may not always be relieved at the end-of-life [8]. Bereaved relatives have reported traumatic experiences of patients’ symptoms not being effectively managed [9, 10].

There is a growing body of evidence considering interventions to manage symptoms including (not limited to) pain, dyspnoea, vomiting, xerostomia, fatigue and agitation for patients with malignant and non-malignant palliative conditions [11–15]. Furthermore, clinical guidelines from the National Institute for Health and Care Excellence (NICE) outline pathways for managing different symptoms for adults in the last days of life: anxiety, delirium and agitation; breathlessness and noisy secretions; nausea and vomiting; supporting hydration [16]. Government policy highlights the need for appropriate and prompt management of symptoms at the end-of-life, to reduce distress for patients and their relatives [17].

In a recent editorial, Higginson [18] highlighted the need for further palliative care research and better utilisation of existing research, following the Neuberger Report [19]. Researchers have raised concerns about the small proportion of research funding allocated to palliative care, particularly in comparison to cancer research [18]. Furthermore, evidence suggests that the research priorities of researchers may not align with those of patients [20, 21], potentially leading to wasted research investment but also patients’ needs not being met [22]. Therefore, Marie Curie and key stakeholder organisations established the Palliative and End of life Care Priority Setting Partnership (PeolcPSP), facilitated by the James Lind Alliance. Surveys with free-text responses have been used successfully within palliative care research to gain detailed insights into patients’ and families’ perspectives [23, 24]. Patients, current and bereaved carers, healthcare professionals, volunteers and members of the public were surveyed about their unanswered questions relating to palliative and end-of-life care. The top 10 research priorities were identified following the James Lind Alliance process, which focused on interventions [25, 26].

Supplementary analysis allows “a more in-depth investigation of an emergent issue or aspect of the data which was not addressed in the primary study” ([27], p.8). The PeolcPSP survey solicited free-text responses, which generated qualitative accounts of respondents’ perspectives and experiences. Following completion of the James Lind Alliance protocol, it was evident that a supplementary analysis would enable analysis of the data set as a whole, including rich data exploring respondents’ experiences that were not associated with interventional treatments.

The aim of this article is to report on a supplementary analysis of the experiences and questions of PeolcPSP survey respondents regarding symptoms, hydration and nutrition.

## Methods

This article has been written according to the Standards for Reporting Qualitative Research (Additional file 1) [28].

### PeolcPSP study design and data collection

The PeolcPSP survey, designed by members of the PeolcPSP team, asked respondents to write responses to two questions (Table 1), identify which category best described them and state where they lived in the UK. The survey ran from December 2013 until May 2014, and was available via a Survey Monkey link widely advertised and in paper format in Marie Curie hospices and nursing services. In total, 1403 completed responses were received. Each individually completed survey was downloaded into NVivo 10 (QSR International Pty Ltd. 2012) as a PDF file from Survey Monkey (San Mateo, California, USA). The paper responses were typed into a word document, checked for accuracy and uploaded onto NVivo 10 (QSR International Pty Ltd. 2012).

**Table 1:** Survey questions

Q. What questions do you have about care, support and treatment of people who are in the last few years of their lives that could help them to live as well as possible? This could also include question(s) about care and support for current carers or families.
Q. What questions do you have about care, support and treatment of people for those rapidly approaching the end of their lives? This could also include question(s) about care and support for current or bereaved carers or families looking after someone at the end of life.

### Supplementary data analysis

An initial coding framework for the supplementary analysis was inductively developed by AN from 200 responses and tested on 50 responses. All 1403 responses were then coded in NVivo 10 (QSR International Pty Ltd. 2012) by a team of qualitative researchers (JB, DA, SS, JVG) using the coding framework, which was adapted as coding progressed to reflect the breadth of the data [29]. The research team (JB, DA, SS, JVG, AB and AN) met weekly during the study period to discuss the coding of the data, whether additional codes had been added to the framework, and – rarely – to resolve any discrepancies through discussion. In total, 190 responses (14%) related to symptoms, nutrition and hydration.

The data relating to symptoms and nutrition/hydration were then analysed thematically by two researchers (JB and DA). Thematic analysis, using Braun and Clarke’s approach, [30] was chosen as it is a flexible approach that can provide a detailed and complex interpretation of the data. This involved:

1. familiarisation with the data through reading and rereading (as described above);
2. generating initial codes using NVivo that described features of the data (as described above);
3. searching for themes and grouping codes into potential themes;
4. reviewing and refining themes;
5. defining and naming themes;
6. producing the written report outlining the themes and final analysis [30].

### Respondents

In total, 190 individual responses related to symptoms and nutrition/hydration. Respondents could choose multiple categories that they felt best described them, e.g., bereaved carer and professional. Therefore, as outlined in Table 2, respondents identified as patients (*n* = 8), current carers (*n* = 24), bereaved carers (*n* = 60), professionals (*n* = 89), volunteers (*n* = 4), members of the public (*n* = 27) and people who selected “other” (*n* = 23). Forty respondents identified in more than one category; the volunteers (*n* = 4) all identified as current or bereaved carers. Fourteen healthcare professionals identified in multiple categories as: a patient (*n* = 1), patient and current carer (*n* = 1), bereaved carer (*n* = 7), current carer (*n* = 4), and a bereaved and current carer (*n* = 1). Of the 12 respondents who selected “other”, 10 identified as a current or bereaved carer. Nine of the current carers also reported as being bereaved.

**Table 2:** Survey Respondents

Respondent (Reporting ID)	Responses relating to symptoms
I am in the last few years of my life (Patient)	8
I am a carer or family member or partner or friend of someone in the last few years of their life (Current carer)	24
I am a bereaved carer or family member or friend (Bereaved Carer)	60
I am a professional working with people in the last few years of life (Professional)	89
I am a volunteer working with people in the last few years of life (Volunteer)	4
I am a member of the public who has an interest in the subject (Member of Public)	27
Other	23
Total	*n* = 235 Individual responses: *n* = 190

### Ethical considerations

Respondents were asked to consent to their participation in the PeolcPSP survey, following a written explanation of the study. The responses were stored on a secure server, only accessible to the research team. Respondents were not asked for identifiable personal information, but responses were anonymised at the point of analysis if respondents included information that could identify them in their responses. Ethical approval was deemed not necessary for the PeolcPSP survey and supplementary analysis by the study sponsor.

### Rigour

The integrity of the supplementary analysis was promoted in three ways. The analysis included the perspectives of multiple groups of respondents, including patients, carers and healthcare professionals, thus increasing the credibility of the study [31]. The data were coded by multiple researchers and the data relating to symptoms were analysed by two researchers (JB and DA), enhancing the trustworthiness of the study findings [32, 33]. Furthermore, the researchers – experienced healthcare professionals or health service researchers - recognised their impact on the research process and sought to be reflexive [34], which was again aided through co-coding and analysis of the data.

## Results

**Fig. 1:**
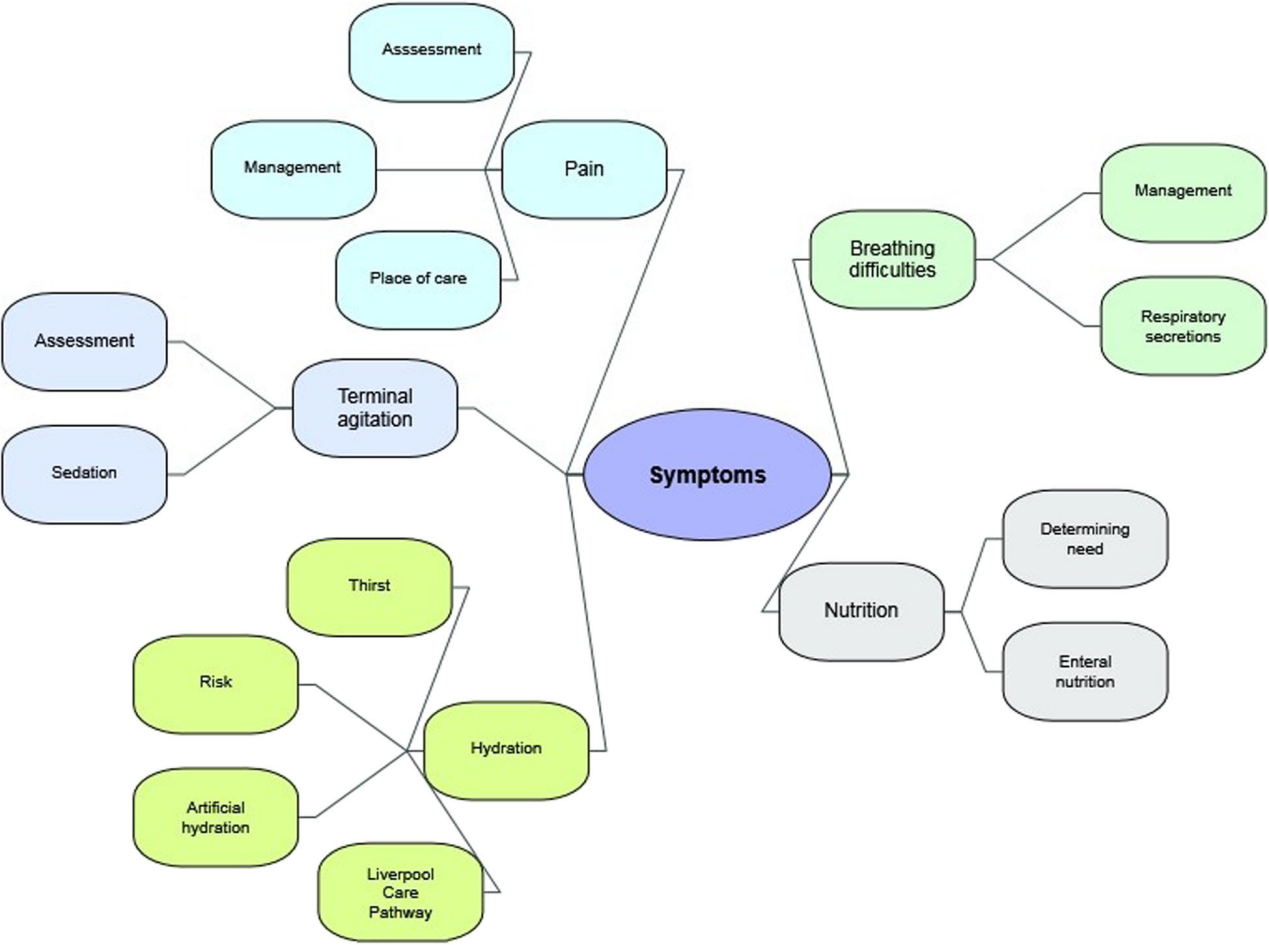
Thematic diagram

Overall, this study identifies that respondents perceive there to be scope and need for improvement in symptom management for individuals at the end-of-life. The following themes and subthemes were identified (see Fig. 1) and are discussed in turn: pain (assessment, management and place of care); breathing difficulties (management and respiratory secretions); terminal agitation (assessment and sedation); nutrition (determining need and enteral feeding); and hydration (thirst, risk, artificial hydration and Liverpool Care Pathway).

### Pain

Pain was the symptom most discussed by respondents. Interestingly, few responses came from people identifying as being in the last few years of life. Primarily responses were from healthcare professionals and current or bereaved carers. Respondents discussed pain assessment, management and the impact of place of care.

#### Assessment

The need for effective pain assessment was highlighted as an important issue by bereaved carers and healthcare professionals. Of note, carers questioned how they would know if their relative (the patient) was in pain, which was viewed as particularly problematic if the patient had a degree of cognitive impairment or were unable to express themselves verbally. Multiple responses related to dementia and concern about how pain can be assessed in patients with this diagnosis:

“How to tell when someone in the very end stages of dementia is in pain and or distress” (R855 - Other - My husband died last year)

Respondents questioned whether methods for assessing pain in people unable to communicate verbally, or with cognitive impairment, were adequate and evidence-based. One healthcare professional, whose son had died from a brain tumour, called for more appropriate methods of assessing pain in people unable to verbally communicate, recognising that pain is a subjective experience:

“How can we assess pain in people who are semi-conscious or under high doses of drugs?…I realise pain can be subjective, but it would be worth looking to develop better pain tools for those who are unable to communicate (either due to level of consciousness, impact of drugs, or due to the condition such as MND or stroke.” (R1064 - Bereaved Carer; Professional)

#### Management

##### Healthcare professional competencies

Respondents questioned whether healthcare professionals would be competent and confident to effectively manage their relative’s pain. Respondents highlighted that pain management was a central aspect of palliative care and their primary concern for people at the end-of-life was that they were pain free. Carer respondents sought reassurance that healthcare professionals would manage their dying relatives’ pain appropriately and ensure they were comfortable:

“How do I know that my relative will be pain free at the end of life, will he/she be properly cared for by professional people” (R1376 – Current Carer)

However, others – primarily bereaved carers - shared their upsetting experiences of their relatives being in pain at the end of their lives. The use of evocative language in the quote below conveys the respondent’s deep feelings about their experience:

“Mummy said to me that why was she suffering so when she had been so good all her life and was this the medieval age as she was being tortured?” (R339 – Bereaved Carer)

Several respondents reflected on the reticence of healthcare professionals to prescribe or administer adequate analgesia as a particular barrier to achieving pain control. While one respondent described nurses refusing to give morphine to their dying relative, another queried why healthcare professionals were seemingly wary of administering analgesia:

“why is it that people who are delegated tasks e.g. pain control are often frightened to do their job - with drugs often late or ineffective?” (R272 – Member of Public)

One bereaved carer described a General Practitioner refusing to prescribe analgesia for their dying mother:

“she was restless, unable to settle and clutching at her chest which made me think she was in a lot of pain. Eventually the staff agreed to call out the on-call GP, who came quickly but said he couldn't give her a pain killing injection as it might kill her, although she was clearly dying; in fact she did die within an hour or two of his visit.” (R812 – Bereaved Carer)

##### Models of care

Respondents offered recommendations for how to improve pain management. A member of the public suggested measures to ensure appropriate, timely pain management for patients. One suggestion included the use of technology such as Skype to enable a healthcare professional to assess a patient without the need for a home visit or the patient attending a clinic:

“pain control needs to be faster, more comprehensive, run by skype, run by experts who can actually prescribe, by people who are not frightened to prescribe and make people comfortable - why is this often not the case?” (R272 – Member of Public)

##### Managing non-malignant pain

Many respondents questioned the most effective ways of managing pain for patients with non-malignant conditions, including motor-neurone disease, Parkinson’s and heart failure. For example, a carer asked:

“What sort of help works best - control of pain and other symptoms, ensuring no restlessness or distress? What is best for those with dementia or heart trouble or other conditions?” (R409 – Current Carer, Bereaved Carer)

#### Place of care

Respondents felt strongly that place of care affected the likelihood of adequate pain management. In terms of hospital care, there was concern that pain relief was not planned for, and patients would not be prescribed adequate levels of analgesia by non-palliative care professionals. A bereaved carer questioned whether non-palliative care professionals need more support to care for patients who are reaching the end of their lives:

“Pain and symptom control is so important, however it is not always delivered in a timely way in hospital. Why do junior doctors find it difficult to prescribe the analgesia in the doses prescribed by the hospice? Do they need more support?” (R1049 – Bereaved Carer, Member of Public)

Conversely, there was unease from other respondents that individuals being cared for at home would not receive effective pain management:

“Support is just not there for people in the last weeks of life for whom medication at home is not adequate to control pain.” (R801 – Bereaved Carer)

Much of the worry about pain management at home related to out-of-hours care provision and whether patients could quickly access analgesia when required; these concerns were reiterated by a palliative care nurse and a patient:

“Why does it still take so long to get someone to come and give pain relief etc. out of hours? The patients should be able to get pain relief etc. very quickly.” (R998 - Other - I am a Marie Curie Nurse)

“how do I deal with things such as nausea, tooth problems and debilitating pain, which can strike at any time (but typically do strike at weekends/pubic holidays)?” (R1165 – Patient)

Place of care was an important issue for carers who lived with feelings of guilt if they were unable to fulfil their relative’s end-of-life wishes. One bereaved carer discussed her feeling of failure that she could not manage her late mother’s pain at home:

“I would have liked her to be able to die at home, that was what she wanted, but I wasn't sure if I could manage her pain and whether getting the Hospice at Home care team there when needed would be feasible. I know I let her down over this.” (R398 – Current Carer, Bereaved Carer)

### Breathing difficulties

Breathing difficulties as a symptom was mentioned less frequently than pain, but was a consistent concern for respondents, who were primarily bereaved/current carers and healthcare professionals. Respondents discussed management of breathing difficulties and respiratory secretions.

#### Management

Respondents questioned the best treatment for breathlessness and discussed the most appropriate time for treatment to commence. One respondent asked when pulmonary rehabilitation should be started for patients with Chronic Obstructive Pulmonary Disease (COPD):

“Not all COPD patients have access to pulmonary rehabilitation despite NICE guidelines, and there is potential to improve their understanding, exercise tolerance and overall progression if targeted at the right time. But when is this?” (R75 - Professional)

Another respondent questioned how support for people with respiratory problems can be improved and whether intervention for breathlessness improves quality of life:

“We currently have no way of measuring if we are having any impact on a patient’s quality of life following input from a physiotherapist, or medical input to manage breathlessness. It would also be beneficial to know if we were able to see patients like this slightly earlier in the disease process, whether we could improve their quality of life for longer.” (R75 - Professional)

#### Respiratory secretions

Respondents asked a series of questions related to terminal respiratory secretions, primarily suggesting that this symptom is poorly managed and asking the reasons for this:

“Why is symptom control of respiratory secretions so poorly managed?” (R1235 – Patient, Current Carer, Professional, Member of the public)

Professionals recognised that this symptom is also upsetting for families:

“Why do we not have effective treatment for the management of respiratory secretions? This problem causes distress for many families who care for and are therefore dealing with this distressing symptom.” (R822 - Professional)

### Terminal agitation

Respondents queried how agitation is best assessed and managed through the use of sedation. One respondent argued for a change in the diagnosis and subsequent treatment of “terminal agitation” through recognising it as “hyperactive delirium”:

“Terminal agitation is a term that has little meaning. Hyperactive delirium at the end of life is a more accurate description. The difference is important since the former is traditionally treated with midazolam while the latter sets in train an assessment and management of the cause and, if drugs are needed, non-sedative haloperidol becomes first choice. An evaluation of end of life hyperactive delirium is long overdue.” (R907 - Professional)

#### Assessment

Several respondents recognised the need for appropriate identification and assessment of terminal agitation, questioning whether biochemical markers can be used to properly diagnose this condition:

“Are there biochemical markers that can help ascertain patients with terminal agitation?” (R1331 - Professional)

#### Sedation

The majority of responses in the agitation theme focused on management, specifically sedation. Carers discussed their negative experiences where sedatives were either not prescribed, or were not effective for their relative. Healthcare professional respondents questioned which sedative was most effective for agitated patients at the end-of-life, and how to ensure adequate doses of sedation are prescribed:

“What is the most effective way to use sedation (e.g. during terminal restlessness) - in order to get the balance right between not giving too much but at the same time giving enough to ease distress.” (R578 - Other - I am a professional now working in another speciality but worked in palliative care between 1997 and 2003)

While respondents recognised the need to treat agitation, there was apprehension about the effect of sedation on the patient. Respondents were worried that carers were not given sufficient information about sedation, which could cause distress. There was also concern that sedation could make communication between the patient and relative difficult, cause nightmares, and hasten death, prompting one respondent to enquire about the effect on the person who has been sedated:

“When people are sedated, are they really unaware of pain/what is being done to them/voices of those they love/extraneous noise from adjacent patients and ward activity? Or are they trapped in a situation where they are aware but cannot tell us? How do we know? How do we know when a person is unconscious rather than sedated?” (R320 – Current Carer, Professional)

### Nutrition

Nutrition was discussed in terms of the longer palliative phase and respondents highlighted the importance of determining patients’ nutritional needs and the role of enteral nutrition.

#### Determining need

Several respondents indicated that further research was required to determine the nutritional needs of people towards the end of their lives. They suggested that identifying nutritional markers would enable healthcare professionals to identify when patients’ nutritional needs are changing. One healthcare professional felt a stronger evidence base would enable carers to feel reassured if the person at the end of their life reduced their dietary intake:

“I have had so many experiences of relatives and professional carers distressed because their loved one/service user hasn't eaten properly. It would be great to be able to re-assure them from the strong position of empirical evidence that their relative is not distressed.” (R1320 - Professional)

#### Enteral nutrition

There were many responses from healthcare professionals querying the role of enteral nutrition for people at the end-of-life. Respondents felt a stronger evidence base was needed regarding if and when enteral nutrition should be administered. Others discussed patients’ information needs and decision-making, including support given to patients to commence and withdraw nutritional support:

“How realistic is the information given to patients regarding PEG feeds… Are they made fully aware that feeding would naturally diminish as the patient deteriorates and that it is therefore not appropriate to be giving 2000 calories in the last weeks/days of life.” (R349 – Professional)

Responses from bereaved carers discussed distressing experiences of enteral nutrition, which highlighted poor communication and lack of respect for patient autonomy. One respondent discussed her father, who had a living will refusing artificial nutrition, being repeatedly asked about having enteral nutrition during the last 4 weeks of his life:

“We found it very hard, because the feeding tube was mentioned again and again, and it was difficult to constantly having to defend his and our decision. The question is: How can health care professionals be persuaded that it is ok not to want a feeding tube and that this is down to patient choice and often better for the patient.” (R687 – Bereaved Carer)

### Hydration

Responses to hydration focused on the last few days of life and considered thirst, risk, the role of intravenous and subcutaneous fluids, and bereaved carers sharing their experiences of hydration and the Liverpool Care Pathway (LCP).

#### Thirst

Several respondents were concerned about patients being thirsty at the end of their lives. One bereaved carer asked whether it is “cruel” not to hydrate patients, while another questioned whether individuals experience a dry mouth or thirst:

“We say that people who do not want to drink at the end of life do not experience thirst, just dry mouth. How do we know?” (R320 – Current Carer, Professional)

#### Risk

Conversely, respondents recognised the risks associated with patients drinking if they have dysphagia. A healthcare professional, who also identified as a bereaved carer, highlighted inconsistent practice, which demonstrated the need for communication between patients, carers and healthcare professionals:

“How to balance providing fluids to those who are dying who cannot swallow safely or easily? The practice of maintaining hydration/nutrition seems variable and inconsistent across patients/hospitals. How can the withdrawal of these be done in a sensitive and consensual way for person, family and medical/caring staff?” (R329 – Bereaved Carer, Professional, Member of Public)

#### Artificial hydration

Following on from these concerns about patients being unable to swallow and thus experiencing thirst, respondents asked about the role of intravenous and subcutaneous fluids. Healthcare professionals questioned whether administration of fluids makes patients more comfortable:

“In the last few days of life families often worry about their loved ones not being given fluids, as a result they are often prescribed subcutaneous fluids. Does this really make the patient more comfortable or not?” (R12 - Professional)

Respondents recognised the concerns of carers and called for further research to identify the support needs of carers when managing artificial hydration for a dying person:

“I think families of dying patients would benefit from research on ways to support them in coming to terms with the withdrawal of IV drips and hydration in the last days of life. I'm convinced this is the source of much dissatisfaction with end of life care.” (R275 – Bereaved Carer)

Another respondent suggested that research is needed to holistically evaluate the role of intravenous fluids for dying patients:

“What are the advantages and disadvantages (physical, social, psychological) of parenteral hydration towards end of life - balancing appropriate hydration with the body's natural ceasing of normal function (also bearing in mind the distress that can be caused when a body cannot cope with increased hydration; the potential for medical ‘kit’ acting as barrier between patient and loved ones towards end of life etc).” (R578 - Other - I am a professional now working in another speciality but worked in palliative care between 1997 and 2003.)

#### Liverpool Care Pathway

Hydration was an emotive subject for bereaved carers, who shared distressing stories of relatives’ deaths, revealing their guilt, anger and sorrow about the Liverpool Care Pathway (LCP). One individual recalled her mother’s death and her residual feelings of guilt that, following the Neuberger Report [19], her mother died feeling thirsty:

“My mother died of breast cancer in the hospice in [names town]. My questions would have been about the Liverpool pathway - it still haunts me whether we did the right thing, and now that it has been stopped, I live with a terrible feeling of guilt that my suspicions were right. It felt wrong to stop fluids but the doctor told me she would effectively drown if they were continued. My mother kept trying to speak to me but was too weak, and I couldn't make out what she was saying. I am so afraid that she was asking for water.” (R398 – Current Carer, Bereaved Carer)

One respondent spoke in even stronger terms about the LCP and described their relative as being “put to death”:

“We as a family have not been able to grieve for our mother who was taken away from us, she was put to death on the LCP and nothing was explained, we were told this is what’s going to happen now!! There was no dignity watching my mother gasp for breath over 4 days, she was denied food and water, why was this.” (R502 – Current Carer, Other - I watched my mother suffer for 4 days on the LCP)

While one respondent questioned how oral fluids could be stopped without an assessment from a speech and language therapist, other respondents asked why their relatives were not given artificial hydration when they could no longer swallow. A bereaved carer asked why the LCP denied artificial hydration, which resulted in them “begging” healthcare professionals for help, highlighting the importance of appropriate communication and engagement with carers at the end-of-life:

“My mother was refused a drip in her final days. As an effect of her brain tumour, she ceased to be able to swallow on 26th December… she was incredibly thirsty and dehydrated but was - despite me begging for help - refused IV fluids even though they would have made her more comfortable. It appears that the Liverpool Pathway specifically denies fluids as part of end of life ‘care’” (R422 – Bereaved Carer)

## Discussion

Undertaking a supplementary analysis of the PeolcPSP data provided a rich insight into the perspectives of 190 patients, carers and healthcare professionals from across the UK. The findings overwhelmingly highlighted that patients, carers, healthcare professionals and members of the public view symptom management as an essential aspect of palliative and end-of-life care. These findings, when located in the broader healthcare context, prompt consideration of evidence-based symptom management, place of care, and specialist/generalist palliative care.

### Evidence-based symptom management

Despite continuing advances in the field of palliative care, symptoms such as pain and breathlessness remain at the forefront of the concerns of clinicians, patients and families [11]. Poorly controlled symptoms have been documented in patients with malignant and non-malignant conditions [35–37], which was reflected in this supplementary analysis.

Bereaved carers in this supplementary analysis expressed concern that pain was under recognised in people unable to verbally communicate, including people with dementia. A recent meta-analysis identified multiple pain assessment tools for patients with dementia, but there was insufficient information on their validity [38]. Furthermore, several non-verbal pain assessment tools have been developed, although a review concluded these tools do not determine level of pain and further research is needed to test the tools with different patient populations [39]. Notably, this supplementary analysis highlights that some carers perceive that the patient’s pain is not being assessed, suggesting that healthcare professionals may not be assessing pain in people with dementia or who are non-verbal, or they are not communicating their assessment to carers. A recent qualitative case study identified that pain assessment tools were not used in practice with patients with dementia, nor were carers included in the pain assessment process [38]. They propose a new decision support tool for hospital-based healthcare professionals to assess pain in patients with dementia [38]. This supplementary analysis highlighted that carers want to know how to assess if their relative is in pain, and further consideration is therefore needed of carers’ role in pain assessment.

This supplementary analysis identified some carers’ concerns that doctors were under-prescribing analgesia, resulting in the patient experiencing pain. Specifically, respondents questioned the wariness of some doctors to prescribe analgesia for their dying relatives, including one respondent who reported a GP’s concerns that he would hasten the death of her mother. Conversely, the Neuberger Report into the LCP highlighted that some carers suspected that the administration of opioids had hastened the death of their relatives [19]. Doctors’ reluctance in prescribing and administering strong analgesics at the end-of-life, due to fear of hastening patient death, has been documented [40]. A recent systematic review of the influence of opioids on survival of advanced cancer patients, showed that there is no evidence associating the use of opioids for symptom control in advanced disease with patient survival [41]. Recently, the British Medical Association (BMA) released guidelines for doctors about the use of analgesia for pain management at the end-of-life, aiming to improve analgesic use [42]. They reiterated that there is insufficient evidence that appropriately prescribed analgesia hastens death but reiterated doctors’ concerns about this.

Unfortunately, many respondents highlighted poor experiences of care where carers’ perceptions were that their relatives were denied food and drinks towards the end of their lives. Eating and drinking is an area that resonates with families due to its familiarity; families may see nutrition and hydration as a basic form of nurturing for their dying relative [43]. Responses in this survey related to the now-withdrawn LCP; the Neuberger Report similarly raised concerns about withholding nutrition and hydration [19]. Guidelines outlining hydration and nutrition at the end-of-life were subsequently developed by the Royal College of Nursing (RCN) [44]; General Medical Council (GMC) guidelines to support decision making were published in 2010 [43]. The impact of these guidelines on practice is unknown at present.

Healthcare professional respondents asserted the need to determine patients’ nutrition and hydration needs at the end-of-life, including whether patients’ nutritional needs diminish as disease progresses, and whether patients feel the sensation of thirst (rather than dry mouth). Respondents argue that establishing answers to these questions would enable healthcare professionals to reassure carers, reducing distress. A recent literature review identified that carers experienced greater distress than patients at reduced nutrition and water intake, leading to attempts at “force feeding” (p. 919) and pressuring their relatives to eat and drink, hoping this would increase survival and quality of life [45]. Artificial hydration and nutrition were viewed positively by these carers [45]. Conversely, this supplementary analysis revealed that some respondents were frustrated when artificial hydration was encouraged against the patient’s or family’s wishes. The Department of Health reports that there remains insufficient high quality evidence regarding assisted nutrition and hydration for patients at the end-of-life [46].

This supplementary analysis demonstrated the necessity for further research into symptom, nutrition and hydration assessment and management. While research was specifically mentioned by healthcare professionals and one carer, other carers asked questions that research may answer. High quality randomised controlled trials (RCT) are critical to test interventions in palliative care, ultimately informing clinical care [47]. Currently, the prevalence and impact of symptoms at the end-of-life are underestimated [48, 49]. Recent RCTs demonstrate the feasibility and necessity for high quality, phase three clinical trials for improving symptom control in this patient population [50–53]. Studies conducted to date have shown that care can be improved [53, 54], patients have a substantial burden of symptoms [49], and that the toxicity and harm of some interventions not underpinned by high quality evidence is underestimated [52, 55]. It is therefore imperative for palliative care to engage further with high quality research.

### Place of care

Respondents had concerns about place of care and whether symptoms, in particular pain, would be better managed in hospital or at home. A large UK survey identified that members of the public associated pain relief with hospital and only 27% of respondents thought they would be pain-free at home at the end of their lives. [54] This was despite 78% of respondents expressing a wish to die at home [56]. A recent systematic review identified that family caregivers viewed hospital as an unsuitable location for palliative care [57]. However, distressing symptoms made home care difficult and, over time, led to hospital being viewed as the preferable option [57]. The Neuberger Report highlighted the concerns of carers that their relatives did not receive adequate and appropriate analgesia in hospital settings at the end of their lives [19]. The recent VOICES survey in England reported that bereaved individuals considered pain management in the last 3 months of life to be more effective in the hospice environment and least effective at home [10]. There are thus conflicting views about which location of care is associated with perceived improvement in symptom management. This was further reflected in this supplementary analysis, which highlighted that respondents were unhappy with pain relief in both home and hospital. Researchers have attempted to establish whether home or hospital is associated with improved symptom control, although results are inconclusive [58]. However, one Cochrane systematic review identified a small, but statistically significant improvement in symptom burden in patients who received specialist palliative care at home [59].

Research consistently concludes that home is the preferred place of care at the end of life for a majority of people with both malignant and non-malignant conditions [60–62], and their carers [57]. Symptoms are one aspect of complex decisions about place of care and this supplementary analysis emphasised that management of symptoms – particularly pain – is a central concern for patients, carers, healthcare professionals and members of the public. It is crucial that high quality evidence around symptom management is established and utilised [19], to ensure that patients’ symptoms are effectively managed, regardless of care location.

### Non-specialist palliative care

Respondents reported dissatisfaction with symptom management by non-specialist palliative care healthcare professionals and questioned whether there was a need for enhanced support to manage symptoms for people with advanced disease. Many patients may not be identified as having palliative care needs, and will therefore not be referred to specialist palliative care teams or specialist palliative care settings at the end-of-life [63]. It is therefore important for increased knowledge transfer of symptom management to both generalists and specialists [64]. A recent review of the current evidence of pharmacological and non-pharmacological interventions for symptom management, produced guidelines for the management of multiple symptoms, aiming to support generalists in the provision of comfort care [65]. Furthermore, in the UK, the NICE pathway outlines symptom management for adults in the last days of life [16].

### Recommendations

Further high quality research for symptom management, including RCTs, is needed and crucially needs to be utilised, to ensure patients’ symptoms are managed across care locations. Furthermore, the role of assisted nutrition and hydration for patients at the end-of-life requires investigation, including patients’ and families’ perspectives. The role of carers in assessing their relatives’ pain needs to be considered, in particular educational support for carers if they are to adopt this role. Finally, the impact of guidelines and responsibilities from the RCN and GMC regarding end-of-life care requires evaluation.

### Limitations

While the researchers were unable to clarify respondents’ reports, or illicit further in-depth information as would be standard in a qualitative interview, the respondents focused on areas of interest to them, without influence from the researchers. Although the researchers were unable to confirm the identity of respondents, due to the anonymous nature of the data, the detailed responses were congruent with individuals who had experience of the phenomena they described. Overwhelmingly respondents identified as being healthcare professionals and current or bereaved carers, with only eight patient respondents who mentioned symptom control. However, the focus of carers’ and healthcare professionals’ responses was the patient and ensuring their symptoms were managed effectively.

## Conclusions

The article has reported on a supplementary analysis of the experiences and questions of the PeolcPSP survey respondents regarding symptoms, hydration and nutrition. Concerns about uncontrolled symptoms and quality of care have been identified from across the respondent groups. Robust, high-quality research investigating the best interventions and medications to manage symptoms will reduce distress for both patients and families, and reduce possible harm of current treatments. Management of symptoms should be equitable across different care settings, to enable patients to remain and die in their preferred place of care. Finally, and possibly unexpectedly, a proportion of healthcare professionals both identified themselves and responded as clinicians, and patients or carers. Palliative care is everybody’s business and the results of this supplementary analysis highlight the need for urgent efforts to improve patient care, sustained by a solid research evidence base.

## Additional file

Additional file 1: Standards for Reporting Qualitative Research [28]. (DOCX 14 kb)

## Abbreviations

BMA: British Medical Association
COPD: Chronic obstructive pulmonary disease
GMC: General Medical Council
LCP: Liverpool Care Pathway
NICE: National Institute for Health and Care Excellence
PeolcPSP: Palliative and end-of-life care Priority Setting Partnership
RCN: Royal College of Nursing
RCT: Randomised controlled trial
UK: United Kingdom
