# Supplementary material for: Symptom management, nutrition and hydration at end-of-life: a qualitative exploration of patients’, carers’ and health professionals’ experiences and further research questions
Source: BMC Palliat Care. 2018 Apr 16;17:60. doi: 10.1186/s12904-018-0314-4 (PMC5901670; doi:10.1186/s12904-018-0314-4)
Supplement: Supplementary file 1 — Standards for Reporting Qualitative Research [28]. (DOCX 14 kb) [file 12904_2018_314_MOESM1_ESM.docx]

**Standards for Reporting Qualitative Research (SRQR) – O’Brien et al. (2014)**

| **No** | **Topic** | **Item** |
| --- | --- | --- |
| S1 | Title | Identifies the topic of the study and the qualitative approach used |
| S2 | Abstract | Abstract includes the necessary sections: background, purpose, methods, results, and conclusions |
|  | **Introduction** |  |
| S3 | Problem formulation | An overview of the literature is provided |
| S4 | Purpose of research question | The purpose of the survey and paper are stated |
|  | **Methods** |  |
| S5 | Qualitative approach and research paradigm | The paper describes that a qualitative approach was chosen, with rationale |
| S6 | Researcher characteristics and reflexivity | The rigour section considers researcher reflexivity |
| S7 | Context | The context of the survey is described |
| S8 | Sampling strategy | The recruitment and sampling processes are outlined |
| S9 | Ethical issues pertaining to human subjects | A section considering ethical issues is included. A statement on ethical approval is also included at the end of the paper |
| S10 | Data collection methods | The methods of data collection are described |
| S11 | Data collection instruments and technologies | The survey questions are outlined in table one |
| S12 | Units of study | A description of the respondents is provided |
| S13 | Data processing | The process of data entry into NVivo is outlined |
| S14 | Data analysis | The thematic approach to data analysis is described |
| S15 | Techniques to enhance trustworthiness | The rigour section considers the integrity of the study |
|  | **Results/findings** |  |
| S16 | Synthesis and interpretation | The findings are presented according to themes identified |
| S17 | Links to empirical data | Data extracts are included throughout the results |
|  | **Discussion** |  |
| S18 | Integration with prior work, implications, transferability, and contribution(s) to the field | The discussion summarises the main findings and explores these in relation to the published literature, identifying the original contribution of this study |
| S19 | Limitations | A section on limitations is included, with reference to the trustworthiness of the findings |
|  | **Other** |  |
| S20 | Conflicts of interest | These are stated at the end of the paper |
| S21 | Funding | This is stated at the end of the paper |
